# Supplementary material for: Refuge distributions and landscape connectivity affect host-parasitoid dynamics: Motivations for biological control in agroecosystems
Source: PLoS One. 2022 Apr 14;17(4):e0267037. doi: 10.1371/journal.pone.0267037 (PMC9009636; doi:10.1371/journal.pone.0267037)
Supplement: S1 File — (PDF) [file pone.0267037.s001.pdf]

SUPPORTING INFORMATION

Refuge distributions and landscape connectivity affect host-parasitoid dynamics: motivations for biological control in agroecosystems

Density time series

Figure S1 shows typical time series for density per site for hosts and parasitoids, for different distributions of refuge areas. The values of density for both species fluctuate around a stationary average that is usually reached after approximately 100 generations.

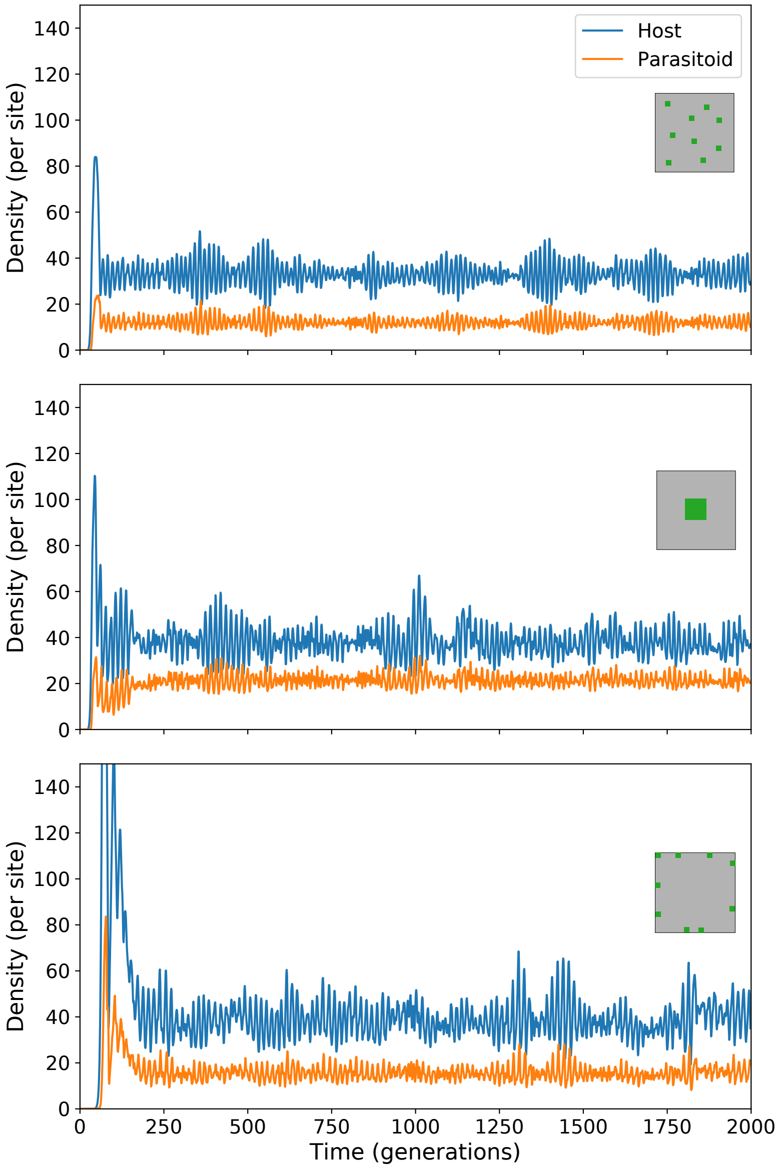

Supporting Figure S1. Time series for densities of hosts (blue - density per crop site) and parasitoids (orange - density per site) for different arrangements of parasitoid refuges: random (top), block(center) and border (bottom). Values for density correspond to a single unit on a ring external network. Coverage of refuge areas corresponds to a fraction of 0.111 of the square lattice.

Statistical analysis

Ring network

Supporting Table S1. Summary statistics for ring network. Summary of T-test statistic for difference between slopes of linear regression models following pairwise comparison of distributions of refuge areas. Differences of slopes are evaluated for host density, host occupancy and Coefficients of Variation (CV), for each parasitoid dispersal radius separately. This table shows the statistics for the ring network.

	Parasitoid dispersal radius							
		Dist. A	Dist. B	Difference (Estimate)	SE	T stat	df	p
Host Density	1	Block	Border	2.23	1.73	1.287	184	0.4043
		Block	Random	32.49	1.03	31.601	184	<0.0001
		Border	Random	30.26	1.73	17.450	184	<0.0001
	3	Block	Border	6.88	2.03	-3.389	184	0.0025
		Block	Random	32.74	1.20	27.204	184	<0.0001
		Border	Random	39.62	2.03	19.519	184	<0.0001
Host Occupancy	1	Block	Border	0.028	0.0027	10.35	184	<0.0001
		Block	Random	-0.069	0.0016	-43.201	184	<0.0001
		Border	Random	-0.096	0.0027	-35.965	184	<0.0001
	3	Block	Border	0.108	0.0120	9.01	184	<0.0001
		Block	Random	-0.218	0.0071	-30.74	184	<0.0001
		Border	Random	-0.326	0.0120	-27.24	184	<0.0001
CV	1	Block	Border	-0.112	0.0277	-4.038	184	0.0002
		Block	Random	0.236	0.0164	14.334	184	<0.0001
		Border	Random	0.348	0.0277	12.537	184	<0.0001
	3	Block	Border	0.044	0.0425	1.034	184	0.5567
		Block	Random	0.357	0.0252	14.166	184	<0.0001
		Border	Random	0.313	0.0425	7.366	184	<0.0001

Star network

Supporting Table S2. Summary statistics for star network. Summary of T-test statistic for difference between slopes of linear regression models following pairwise comparison of distributions of refuge areas. Differences of slopes are evaluated for host density, host occupancy and Coefficients of Variation (CV), for each parasitoid dispersal radius and each type of node separately. This table shows the statistics for the star network.

	Node	Parasitoid dispersal radius							
			Dist. A	Dist. B	Difference (Estimate)	SE	T stat	df	p
Host Density	Central	1	Block	Border	-6.51	3.62	-1.798	13	0.208
			Block	Random	24.78	2.15	11.54	13	<0.0001
			Border	Random	31.3	3.62	8.639	13	<0.0001
	Periferic	1	Block	Border	26.6	4.53	5.859	165	<0.0001
			Block	Random	46.5	2.69	17.311	165	<0.0001
			Border	Random	20.0	4.53	4.405	165	0.0001
	Central	3	Block	Border	-21.9	5.36	-4.088	13	0.0034
			Block	Random	12.9	3.18	4.044	13	0.0037
			Border	Random	34.8	5.36	6.486	13	0.0001
	Periferic	3	Block	Border	21.2	5.11	4.153	165	0.0002
			Block	Random	39.4	3.03	13.02	165	<0.001
			Border	Random	18.2	5.11	3.566	165	0.0014
Host Occupancy	Central	1	Block	Border	0.0277	0.0057	4.804	13	0.0009
			Block	Random	-0.024	0.0034	-7.013	13	<0.0001
			Border	Random	-0.0517	0.0057	-7.013	13	<0.0001
	Periferic	1	Block	Border	-0.0731	0.0076	-9.611	165	<0.0001
			Block	Random	-0.1356	0.0045	-30.08	165	<0.0001
			Border	Random	-0.0626	0.0076	-8.228	165	<0.0001
	Central	3	Block	Border	0.187	0.039	4.785	13	0.0010
			Block	Random	-0.0742	0.0231	-3.206	13	0.0176
			Border	Random	-0.261	0.039	-6.686	13	<0.0001
	Periferic	3	Block	Border	-0.3807	0.0364	-10.447	165	<0.0001
			Block	Random	-0.4305	0.0216	-19.926	165	<0.0001
			Border	Random	-0.0498	0.0364	-1.368	165	0.3602
CV	Central	1	Block	Border	-0.0816	0.0707	-1.155	13	0.4992
			Block	Random	0.252	0.0419	6.024	13	0.0001
			Border	Random	0.3341	0.0707	4.727	13	0.0011
	Periferic	1	Block	Border	0.0104	0.0425	0.244	165	0.9678
			Block	Random	0.3279	0.0252	13.000	165	<0.0001
			Border	Random	0.3176	0.0425	7.464	165	<0.0001
	Central	3	Block	Border	-0.325	0.0923	-3.520	13	0.0098
			Block	Random	0.284	0.0547	5.196	13	0.0005
			Border	Random	0.609	0.0923	6.601	13	<0.0001
	Periferic	3	Block	Border	0.789	0.0659	11.973	165	<0.0001
			Block	Random	0.542	0.0391	13.863	165	<0.0001
			Border	Random	-0.247	0.0659	-3.753	165	0.0007
