# Supplementary material for: Refuge distributions and landscape connectivity affect host-parasitoid dynamics: Motivations for biological control in agroecosystems
Source: PLoS One. 2022 Apr 14;17(4):e0267037. doi: 10.1371/journal.pone.0267037 (PMC9009636; doi:10.1371/journal.pone.0267037)
Supplement: Attachment — Submitted filename: response_to_reviewers.pdf [file pone.0267037.s002.pdf]

Refuge distributions and landscape connectivity affect host-parasitoid dynamics: motivations for biological control in agroecosystems

(PONE-D-21-33487)

Reply to the Reviewers

(Dated: February 1, 2022)

DETAILED REPLY TO THE REPORT OF REVIEWER 1

COMMENT 1.1

I definitely see the relevance of thinking of different spatial scales of landscape, and scales of movement influencing host-parasitoid interactions, their abundances and dynamics. But I don't see how the scales presented here correspond to agricultural landscapes. Figure 1 shows small scale areas with refuge imbedded in it (individual farms), connected together on a larger in ring and star configurations. What does the area between the farms represent? Why that configuration? What is the difference between that and "non-crop" habitat which is called refuge when it is in the farm?

Reply 1.1.—We thank the reviewer to see the relevance of our approach.
In fact, our main goal when choosing a representation of the landscape with different layers of networks was to explore some of the main features that can be found in real agricultural landscape, investigating how these features can affect population dynamics, particularly focusing on pest control. The two scales we investigate then represent local dispersal, within the same farm, and regional dispersal, between different farms. One important motivation for exploring these two scales is that strategies of biological control can be less effective if applied only in a chosen farm, while ignoring the regional structure where this farm is located. An integrated approach to pest control thus requires understanding not only how species spread within the unit of production but the likelihood of pests and natural enemies being exchanged between different units.

The choice of ring and star networks to represent a regional setting, while admittedly a simplification of reality, allows the investigation of how the structural connectivity of a production unit in the regional scale (how many different units it is connected to) affects population dynamics within it. Ring and star networks thus represent two extremes of connectivity scenarios: one where every unit has the same connectivity pattern (ring) and another where one of the units has a drastically different pattern from the others (star).

We defined the parasitoid non-crop refuges as "patches where it is possible to find alternative hosts and food supplies, allowing reproduction and maintenance of a small population of parasitoids". Thus, in our model, the space outside the farms can represent urban areas, freshwater or any form of land use not suitable for the species we consider. While a more complex spatial representation is possible, allowing for this between-farm space to be used by species, we wanted to focus on two specific spatial features: landscape connectivity and within-farm composition. This was the main reason for the adoption of this spatial representation.

We have altered the first three paragraphs of the Methods section to try to make the points raised by the reviewer clearer. The justification for the regional structure can now be seen in the paragraph:

"The choice of these two arrangements highlights different spatial scenarios that might be imposed by regional topography: homogeneous connectivities, where all production units have the same number of neighbors, and heterogeneous connectivities, where the central unit behaves as a hub and the other units have only one link. The space between these units can represent urban areas, freshwater or any form of land use not suitable for the species we consider. While admittedly a simplification of reality, this representation enables the exploration of how differences in the structural connectivity of production units influence the dynamics of pests and natural enemies locally, emphasising the importance of integrated regional approaches to deal with common threats".

Non-crop and refuge patches are also better explained in this revised version. Please refer to Reply 2.2 for detailed descrip-

tion.

COMMENT 1.2

> *Since there are more important generalist predators than parasitoids in agricultural systems, it may make sense to change the language in the ms to be about predator-prey rather then host-parasitoid dynamics.*

Reply 1.2.—While we acknowledge the importance of generalist predators in strategies of pest control (particularly on systems of conservation biological control), such a change on the focus of the study would lead to a different set of models, with distinct mathematical formulation and dynamics.

Parasitoids are important organisms for strategies of biological control, both augmentative and conservation control, and the degree to which they can be considered specialists or generalists depends a lot on the context. For exemple, parasitoid wasps of the genus *Trichogramma* are commonly employed for biological control and are known to target many different species of Lepidoptera. A degree of generalist behaviour can also be found in different species of wasps considered natural enemies of agricultural pests.

Therefore, we argue that the choice of a host-parasitoid system is justified, given our goal to explore the interplay between species interactions and landscape structure in agroecosystems. The additional investigation of predator-prey systems in the same context would certainly enrich this knowledge, but it lies outside the scope of the present work.

COMMENT 1.3

> *I am not qualified to evaluate the novelty of the model, but I will note that it wasn't discussed in relation to other spatial host-parasitoid dynamics models. Thus maybe it is difficult to assess the contribution of this particular model and set of simulations. Perhaps analysis of local and regional scales simultaneously is novel, but there has been significant modeling of the two scales separately. The value of this study would be increased by placing it more the context of other work.*

Reply 1.3.— We agree with the reviewer that our manuscript could be improved by better contextualising it with previous work in the area.

While recognising the existence of an extensive literature on theoretical models in Ecology and, in particular, models exploring host-parasitoid dynamics, we also note that to the best of our knowledge, no previous study has investigated how local and regional scales, treated simultaneously, influence species interactions and distributions, as the reviewer also acknowledges on their comment.

Furthermore, while discussing our results, we emphasised possible consequences for real scenarios, establishing a relationship with landscape ecology, instead of focusing on the analysis of metapopulation dynamics, for which many examples can be found in the literature. By doing so, we also intended to reach the broader audience of PLOS One that might be interested on the implications of the results to agroecosystems.

Following the reviewer's suggestion of contextualisation with previous work, we have included the following paragraph on the Introduction:

"In the attempt to understand how different landscape features influence species interactions and distributions, mathematical and computational models have long been recognised as important tools. Considering more specifically host-parasitoid interactions, many studies were conducted to analyze stability, persistence, spatial dynamics in parasitoid communities, and theoretical approaches to diffusion. Although these studies have significantly contributed to our understanding of spatial aspects of host-parasitoid models, none of them investigated the effects of simultaneously considering different spatial scales on insect movement, nor its implications for the effectiveness of biological control in agroecosystems."

COMMENT 1.4

Overall, I found the introduction and abstract unclear, but the results and discussion pretty clear. Though the discussion was clear in could benefit from more reference to related work from the literature.

Reply 1.4.—We thank the reviewer for pointing out the manuscript's weaknesses and for highlighting its strengths. We changed the abstract according to reply 1.5 and modified the text to make the introduction clearer. We also added a paragraph in the Introduction with reference to related work from the literature and further motivation for our work (please also see reply 1.3).

COMMENT 1.5

Specific comments:
Abstract:
- Consider leaving out the second sentence
- What do you mean by non-crop habitat? Do the hosts not inhabit it? Later I see what you mean, but in the abstract it is confusing.
- The abstract will be more interesting with specific outcomes of the study stated, so what are some "responses of host suppression"? And, how does parasitoid dispersal range interact with landscape structure to affect host population variables?

Reply 1.5.—We thank the reviewer for their suggestions to the abstract. We made all the changes accordingly.

COMMENT 1.6

Line 15-16: If you are sticking with the host-parasitoid set up rather than predator-prey, consider introducing the work in the context of other spatial models of host-parasitoid dynamics such as C. Briggs, W. Murdoch, J. vandermeer, P. Amarasekare, Susan Harrison

Reply 1.6.— We have now included additional reference to related literature, also improving the motivation of our work in this context. Please refer to Reply 1.3 for a detailed description.

COMMENT 1.7

Line 20, references 16 and 17: Again, these don't seem like specific references for this idea.

Reply 1.7.— We agree with the referee. We replaced the old references by other ones more appropriate to the text ideas.

COMMENT 1.8

Line 23 references: These references are more about predators than parasitoids.

Reply 1.8.— In this version of the manuscript we used a reference specific for generalist parasitoids. Thank you.

COMMENT 1.9

Line 30: missing the word "viable"?

Reply 1.9.— We have included this suggestion. Thank you.

COMMENT 1.10

Line 38: "Guarantee" sounds pretty absolute. How about facilitate? or promote?

Reply 1.10.— We have included this suggestion. Thank you.

COMMENT 1.11

Line 41: Connectivity is defined by different people in different ways. Sometimes connectivity is a function of the distribution of the habitat and the dispersal ability of the species, so it isn't independent of dispersal.

Reply 1.11.— We thank the reviewer to point this out. In this work, we focus on structural connectivity (physical configurations and spatial features of the landscape) instead of functional connectivity, that would take into account biological and behavioural aspects of the species. We have included this explanation on the text to make it clearer:

"Here we investigate how the interplay between dispersal at different spatial scales and landscape structural connectivity and composition (in other words, physical configurations and spatial features) affect species dynamics and distributions."

COMMENT 1.12

Line 43: Is two distinct scales a general pattern? I don't quite see it. I can imagine movement within a patch that isn't really dispersal, and then dispersal, that could be at a range of scales, but not two distinct ones. Am I missing something?

Reply 1.12.— As described on the manuscript, each site on the square lattice corresponds to a patch, "formed by a small group of individual plants". We consider that within-patch movement is implicit in the discrete-generation dynamics of interaction.

Dispersal, therefore, is a different process, happening after the interaction stage and only once per generation (please, refer to Methods for detailed description). It can happen in two scales: a local one, where individuals disperse to nearby patches on the same square lattice (within the area of one dispersal radius), and a regional one, with dispersal to adjacent square lattices (if linked on the regional structure).

COMMENT 1.13

Line 47: Define what you mean by non-crop. How is that related to the term "refuge" as you use it?

Reply 1.13.— We have now clarified the meaning of "non-crop" and "refuge" on this paragraph and improved this explanation on the Methods. Please refer to Reply 2.2 for the motivation and complete description of the changes.

COMMENT 1.14

Line 51: What are the host variables?

Reply 1.14.— The host variables are density and occupancy, as presented on item (i) on this paragraph. We used this construction to avoid repetition on item (ii).

COMMENT 1.15

Line 53: What is connectivity in this case, and the dynamics of which species? And the spatial dynamics or population growth rate dynamics?

Reply 1.15.— For the point raised on connectivity, we have added a note on the previous paragraph. Please see Reply 1.11.

We focus on the changes in population variables of the host species, but those can only be understood by considering how parasitoids might also be affected on the different scenarios. For this reason, we use "species dynamics" without specifying.

COMMENT 1.16

Line 58-59: I didn't get from the introduction how these two discrete scales correspond to in real landscapes.

Reply 1.16.— Local and regional scales are briefly described in the Introduction at:

"On the local scale, species interact on a landscape with regular structure and short distances between patches. On the regional scale, different landscapes are interconnected by dispersal through comparatively larger distances."

Then on subsection "Spatial structure" in the Methods section further details of the local and regional scales are presented and justified. Following the reviewer's concerns, we have altered the initial paragraphs of this section to try to make these points clearer. Please refer to Reply 1.1 for a detailed explanation.

COMMENT 1.17

Line 61-63: This should be said earlier. Still though, I don't see how this is two scales. Is there refuge or crop or something else between farms?

Reply 1.17.— Please refer to Reply 1.1 and Reply 2.2.

Comment 1.18

Line 95: Density dependence of what?

Reply 1.18.—Density dependence affects only hosts. To make it clearer, we have changed this sentence for:

"with parasitoids that attack hosts' eggs and host density-dependent effects that become relevant at the larval stage"

Comment 1.19

Line 162-163: Why a star and a ring?

Reply 1.19.—As we mentioned in Reply 1.1 we model the external network representing the regional scale of an agricultural landscape, using two different connectivity patterns: the ring and the star network. We choose these two arrangements to highlight different spatial scenarios that might be imposed by regional topography: homogeneous connectivities and heterogeneous ones. Both constitute one of the most simplified representations of such structures. But despite this simplification of reality, each representation enables the exploration of how differences in the structural connectivity of production units influence the dynamics of pests and natural enemies locally. This choice is now better explained in the Spatial Structure part of the Methods section.

Comment 1.20

Line 175-176: Explain the Parameters briefly so that the reader understands generally without looking at another paper.

Reply 1.20.—We have now introduced the following:

"Parameters for the interaction and dispersal stages, in particular maximum dispersal rates and tolerances of hosts and parasitoids to conspecifics at the same patch (see complete description on Methods), were obtained from a previous host-parasitoid experimental setting."

Comment 1.21

Line 186: What are these traits and how were they chosen?

Reply 1.21.—We have changed that to the more specific "parasitoid dispersal ranges".

We thank the reviewer for their thorough evaluation of our work. In this new version, we took into account the reviewer's comments, which indeed improved our manuscript.

DETAILED REPLY TO THE REPORT OF REVIEWER 2

COMMENT 2.1

This contribution explores the dynamics of host-parasitoid systems in connected patches based on different topologies. I think the paper is well written and the topic of spatial effects in quite pertinent to these systems. However, some discussion around these points is missing in the paper:

Reply 2.1.— We thank the reviewer for the comment that the "the paper is well written and the topic of spatial effects in quite pertinent to these systems." Below, we answer in detail the concerns raised by the reviewer. We hope that our answers will satisfy his/her criticisms and comments.

COMMENT 2.2

1) The concept of refuge has to be motivated and discussed. Generally, refuges imply a host refuges where parasitoids are unable to attack hosts, but I found this concept of "parasitoid refuge" confusing. What is the motivation for a parasitoid refuge and how is that different from host refuge?

Reply 2.2.—We thank the reviewer for making this important point. Although we briefly mention parasitoid refuges in the Introduction, the concept we use in our manuscript was only made clear on the Discussion:

"In our model, refuge areas correspond to non-crop patches where it is possible to find alternative hosts and food supplies, allowing reproduction and maintenance of a small population of parasitoids".

Particularly in the context of conservation biological control, the existence of alternative hosts and food supplies is fundamental for the persistence of natural enemies in a given area, increasing the effectiveness of the control. In this sense, the term refuge in our manuscript is different from the more common concept of host/prey refuge, where an organism obtains protection from predation by hiding. However, given its importance for biological control in agroecosystems, and treating the term refuge as a more general spatial feature that allows increased survival of individuals, we decided to expand its meaning and refer to these non-crop patches as parasitoid refuges.

To make this choice clearer we have included on the Introduction:

"Each of these landscapes is characterized by different distributions of non-crop habitats, where hosts are unable to grow and parasitoids are provided with alternative hosts. We call these habitats parasitoid refuges, directly influencing parasitoids' spatial distributions and, consequently, their interaction dynamics with the primary hosts."

And then, expanded on the Methods:

"Patches on the lattice can be of two types: crop or non-crop areas. We assume hosts to be specialists on that specific crop and, therefore, unable to grow on non-crop areas. Additionally, non-crop areas are also assumed to provide alternative hosts and food supplies for parasitoids, allowing reproduction and maintenance of small populations, and we refer to them as refuges. Note that this is different from the more common concept of host/prey refuge, where an organism obtains protection from predation by hiding. However, treating refuges as more general spatial features that allow increased survival of individuals (in this case, parasitoids), we use this term henceforth."

Comment 2.3

2) Connected to the earlier point, host refuges are critical to stabilize host-parasitoid systems So the authors should include them in the simulations to see how that the overall dynamics and results are altered.

Reply 2.3.—The reviewer is right to point out that host refuges are known to stabilize host-parasitoid systems, however they are not the only features able to do so. Spatially-explicit dynamics and density-dependent effects are also know to stabilize host-parasitoid systems and indeed this is the result we obtain in our work. After an initial transient of population expansion and decline, host and parasitoid populations fluctuate around a stable average level for each of the scenarios we have studied.

We have now included plots for some of the time-series we have encountered showing post-transient stability of the populations. The new figures can now be seen in the Supporting information (Figure S1).

Comment 2.4

3) The authors mention dynamics but there no time-traces of population densities over time. It will be good include these to see what the actual dynamics looks like.

Reply 2.4.—Please, see Reply 2.3. The new figures can now be seen in the Supporting information (Figure S1).

Comment 2.5

4) The Nicholson Bailey models is unstable resulting in diverging oscillations. Generally, I found a lot of this context on stability missing in the entire papers. I could not find the stability analysis for these systems to see if they are indeed stable or not. Can the authors present this analysis?

Reply 2.5.—As mentioned before (please see Reply 2.3) several mechanisms are known to stabilize population dynamics in the Nicholson-Bailey model. In particular, our model considers spatially-explicit dynamics and density-dependency on hosts, leading to small fluctuations around a stationary population level after an initial transient. We have now included new figures on the Supporting information showing typical examples of the time-series for hosts and parasitoids.

A complete stability analysis, although valuable and interesting on its own, goes beyond the scope of the present work. Here, instead of focusing on the thorough investigation of the dynamical phenomena on this system, we intended to discuss the implications of the spatial structure for the biological control on agroecosystems. Therefore, the new figures in the Supporting information (Figure S1) should be enough to convince the reader that the time averages we use are consistent.

Comment 2.6

5) This recent paper came up in my search on spatial effect and seems relevant to this study. It will be good discuss and compare: https://www.sciencedirect.com/science/article/abs/pii/S0025556420300869

Reply 2.6.—Indeed, this reference is very interesting and we thank the reviewer for showing it. However, in this work, the authors characterized parameter regimes leading to stable and unstable population dynamics, considering different forms of spatial heterogeneity. As we mentioned in replies 2.3 and 2.5, this type of stability analysis, although interesting on its own, is outside the scope of this work.

In this revised version we have also included new references to bring our work closer to the context of theoretical approaches to metapopulation dynamics. Please refer to Reply 1.3 for a complete description.

We thank the reviewer again for their thorough evaluation of our paper. We implemented the reviewer's suggestions, clarifying important points of the manuscript and improving our paper.
