# Supplementary material for: Refuge distributions and landscape connectivity affect host-parasitoid dynamics: Motivations for biological control in agroecosystems
Source: PLoS One. 2022 Apr 14;17(4):e0267037. doi: 10.1371/journal.pone.0267037 (PMC9009636; doi:10.1371/journal.pone.0267037)
Supplement: Attachment — Submitted filename: response_to_reviewers_R02.pdf [file pone.0267037.s003.pdf]

Refuge distributions and landscape connectivity affect host-parasitoid dynamics: motivations for biological control in agroecosystems
(PONE-D-21-33487R1)

Reply to the Reviewer

(Dated: March 30, 2022)

DETAILED REPLY TO THE REPORT OF THE REVIEWER

COMMENT 1.1

The authors have answered satisfactorily all the questions raised by the reviewers and the editor in this new version. The only minor change I request is to modify a sentence that currently does not make sense grammatically:

L101-102: However, treating refuges as more general spatial features that allow increased survival of individuals (in this case, parasitoids), we use this term henceforth.

Please note also that the connection with the use of 'refuge' is lost, I think, by the previous sentence. Once you change this sentence, please resubmit the new version.

Reply 1.1.—We thank the reviewer for the thorough evaluation of our manuscript. We have corrected the previous sentence, better explaining our use of the term refuge, which now reads:

"Note that this is different from the more common concept of host/prey refuge, where an organism obtains protection from predation by hiding. *Here, the term refuge is used as a generalisation, referring to spatial features that allow increased survival of individuals, which, in our model, correspond only to parasitoids finding alternative hosts and resources at these locations. Henceforth, we use refuge as reference to parasitoid refuge in this context.*"
